# A patient-initiated treatment model for blepharospasm and hemifacial spasm: a randomized controlled trial

**DOI:** 10.1186/s12883-022-02603-7

**Published:** 2022-03-17

**Authors:** Sadie Lawes-Wickwar, Hayley McBain, Stefano Brini, Shashivadan P. Hirani, Catherine S. Hurt, Chris Flood, Nicola Dunlop, Dianne Solly, Bridget Crampton, Stanton P. Newman, Daniel G. Ezra

**Affiliations:** 1 City, University of London, School of Health Sciences, 10 Northampton Square, London, EC1V 0HB UK; 2 University College London, Institute of Epidemiology and Health Care, London, UK; 3 London South Bank University, School of Health and Social Care, London, UK; 4 Moorfields Eye Hospital NHS Foundation Trust, Adnexal Department, London, UK; 5 Public Contributor, London, UK

**Keywords:** Patient-initiated services, Randomized controlled trial, Blepharospasm, Hemifacial spasm, Dystonia, Botulinum toxin

## Abstract

**Background:**

To test, in a two-arm, single center, superiority, randomized controlled trial, the effectiveness of and costs associated with a patient-initiated treatment model for people with hemifacial spasm (HFS) and blepharospasm (BEB) in comparison to usual care.

**Methods:**

One hundred and thirty patients with HFS or BEB, aged 18 years or over, were recruited from a nurse-led botulinum toxin type A clinic at an eye hospital in the United Kingdom (UK), completed baseline measures and were randomized (1:1). The intervention group determined their own botulinum toxin type A (BoNT/A) treatment schedule during the trial period (9 months) and received an information leaflet with a “hotline” number to book an appointment. Usual care appointments were scheduled by treating clinicians. Data analysts were blind to study group. The primary outcomes were disease severity and functional disability, as measured by the Jankovic Rating Scale and Blepharospasm Disability Index, respectively. Secondary outcomes included quality of life, anxiety and depression, satisfaction with care, confidence in the service, economic costs and employment days lost.

**Results:**

Sixty-five patients were randomized to each group. The intervention demonstrated no statistically significant difference to usual care for any of primary outcomes. On secondary outcomes the levels of anxiety differed significantly (F_2, 142.39_ = 1.65, *p* = 0.02), with the intervention arm exhibiting a decrease and the control arm an increase (Hedges’ g = − 0.26 [99% CI -0.83, 0.32]). No other statistically significant differences were found for secondary outcomes. Overall healthcare costs and costs to the patient were on average £198.95 less (95% CI -£256.76, £654.67; *p* = 0.10) per participant for those in the intervention compared to usual care, although this finding was not significant.

**Conclusions:**

We did not observe differences between the patient-initiated treatment model and usual care for people with BEB or HFS, on any primary outcome measure, quality of life, or depression. The patient-initiated treatment model may, however, have the potential to save healthcare costs and reduce anxiety. Patients using this new model were also equally as satisfied in the service and confident in their care as those receiving treatment as usual.

**Trial registration:**

Clinicaltrials.gov ID NCT02577224, 16th October 2015.

**Supplementary Information:**

The online version contains supplementary material available at 10.1186/s12883-022-02603-7.

## Background

The primary treatment for benign essential blepharospasm (BEB) and hemifacial spasm (HFS) is botulinum toxin type A (BoNT/A), which results in a fluctuating pattern of relief and symptom return [1]. Patients report wide ranging benefit from repeated treatment [2]. Despite this variation in patients’ experiences, a standardized treatment regimen of injections on average 3 times a year is typical throughout the United Kingdom (UK). This may mean that some people experience debilitating symptoms for several weeks until their next appointment and some are seen too often. Alternative models of care, offering individualized treatment plans, therefore need to be considered.

Personalized care is central to the National Health Service (NHS) Long Term Plan [3], where patients obtain greater control over their condition and treatment. Patient-led healthcare services have the potential to address the variability in patients’ experiences of the current BoNT/A treatment regimen. Patient-initiated treatment models for chronic conditions, where the patient has control over treatment timings to manage their symptoms, are associated with no significant differences in psychological or health-related quality of life [4] or clinical outcomes [5] compared to standard care. The risk of harm is therefore deemed low, and, in some instances, there are also savings in time and costs [5]. Although patient-initiated treatment models have been implemented in dystonia, there is no evidence for their effectiveness in a real-world setting. This study aims to evaluate a patient-initiated treatment model for people with BEB or HFS receiving BoNT/A, in terms of clinical impact and associated costs.

## Methods

Full details of the study protocol have been reported previously [6]. The trial followed the Consolidated Standards of Reporting Trials (CONSORT) statement [7].

### Study objectives

The primary objective was to establish whether a patient-initiated treatment model for those with BEB or HFS was associated with reduced disease activity or functional disability in comparison to usual care.

### Trial design

The study was a two-arm, single center, superiority randomized controlled trial (RCT). Participants were randomized to receive either (1) standard care, or (2) the patient-initiated treatment model. Randomisation (1:1) was performed by a Senior Data Manager in the Research and Development Department at Moorfields Eye Hospital (MEH) NHS Foundation Trust, London, in order to ensure allocation concealment. Randomly permutated blocks of varying sizes ensured balance between treatment groups on sample size, preventing serious imbalance in sample size should the study have been terminated prematurely. Group allocation was concealed from the researchers who performed statistical analyses and only revealed after all analyses had been completed.

### Participants

Participants were recruited from a twice-weekly nurse-led BoNT/A treatment clinic at MEH, between August 2015 and February 2017. Participants were recruited by a health psychology researcher and research nurse. Eligible participants (aged 18 years or over) had been diagnosed with HFS or BEB by a consultant. The decision to include BEB and HFS was due to overlapping symptomology and equivalent treatment protocols between these patient groups. All patients were on a stable dose of BoNT/A, defined as having received the toxin over two previous cycles and free from treatment-related side effects including ptosis, double or blurred vision, and foreign body sensations, as recorded in their hospital notes. This screening took place so randomisation to the patient-initiated appointment group thereafter did not conflict with any necessary ongoing monitoring (e.g., follow up appointments scheduled by the specialist nurse). Patients were excluded if they had significant comorbidities (i.e., their predominant treatment was for another illness), and/or were unable to communicate fluently in English to complete study measures. Participants were randomized to one of two treatment conditions after providing informed consent to participate in the study and returning a baseline assessment.

### Intervention group

Participants randomized to the intervention group determined their own BoNT/A treatment schedule during the trial period (9 months) and were given an instruction leaflet and contact details for a dedicated booking line. Participants contacted the nurse-led clinic via the booking line when they felt their symptoms returning All patients with a disease activity score of 1 or above on the Jankovic Rating Scale (JRS) [8] were booked into the next available clinic slot. Although there was no limit the number of times participants in the intervention group could initiate an appointment, participants were advised to wait at least 2 weeks after treatment as this is the maximum time it takes for botulinum toxin to become effective.

### Control group

Participants in the control group received usual care, whereby each follow-up appointment was scheduled by their treating clinician based on their historical patterns of treatment.

### Measures

An initial baseline assessment was taken prior to randomization, this included the full range of measures described below, clinical variables and demographic information.

#### Primary outcome measures

The clinician-reported JRS [8] was measured at baseline and 9 months for all trial participants. The patient-reported Blepharospasm Disability Index (BSDI) [9, 10] was measured at baseline and again after 3 and 9 months, again for all trial participants.

#### Secondary outcome measures

The Client Satisfaction Questionnaire (CSQ) [11], Craniocervical Dystonia Questionnaire (CDQ-24) [12], Hospital Anxiety and Depression Scale (HADS) [13] and a 10-point visual analogue scale measuring confidence in the service were measured at baseline and again after 3 and 9 months. Use of health and social care services was estimated using a brief version of the Client Service Receipt Inventory (CSRI) [14] completed by a combination of self-report and electronic patient records at the end of the 9-month trial. Unit costs of healthcare resources were derived from the NHS trust where possible and national unit costs [14–18] where this was not possible.

### Statistical methods

Preliminary analyses provided descriptive details for all the measures at the item or scale level, as appropriate. Little’s Missing Completely at Random (MCAR) test indicated that the data were MCAR (*p* > 0.05). In turn, we imputed the missing data using multiple imputation. Ten scale-level imputation iterations were used as recommended [19]. This dataset was used to explore differences between groups at baseline and for differences in the economic and resource use at the end of the trial using independent samples t-tests. These analyses were repeated after removal of outliers, defined as 2 standard deviations or more from the mean. The results presented exclude these outliers.

We explored the stability and changes in the outcome measures across the three time points using multi-level modelling (MLM). Trial arm, time and the interaction between trial arm and time were entered as fixed effects in each model, with participant identification number as a random effect. Standardized adjusted effect sizes for group differences at each time point were calculated using Hedges g along with 99% confidence intervals using the appropriate formula [20]. These effect sizes were interpreted in the same way as Cohen’s d [21] (small = 0.20, medium = 0.50, large = 0.80). For all tests a *P* value was considered significant at 0.05.

### Sample size

The sample size requirement for the study was estimated at. between 112 (56 per group) and 278 (139 per group) participants, based on a 79% completion rate [22].

### Classification of evidence

The primary research question was whether a patient-initiated treatment model for those with BEB or HFS was associated with a deterioration in disease activity or functional disability in comparison to usual care. This study provides Class I evidence that for patients with HFS or BEB there were no significant differences in disease activity or functional disability when patients were able to initiate their own treatment in comparison to treatment as usual.

## Results

**Fig. 1:**
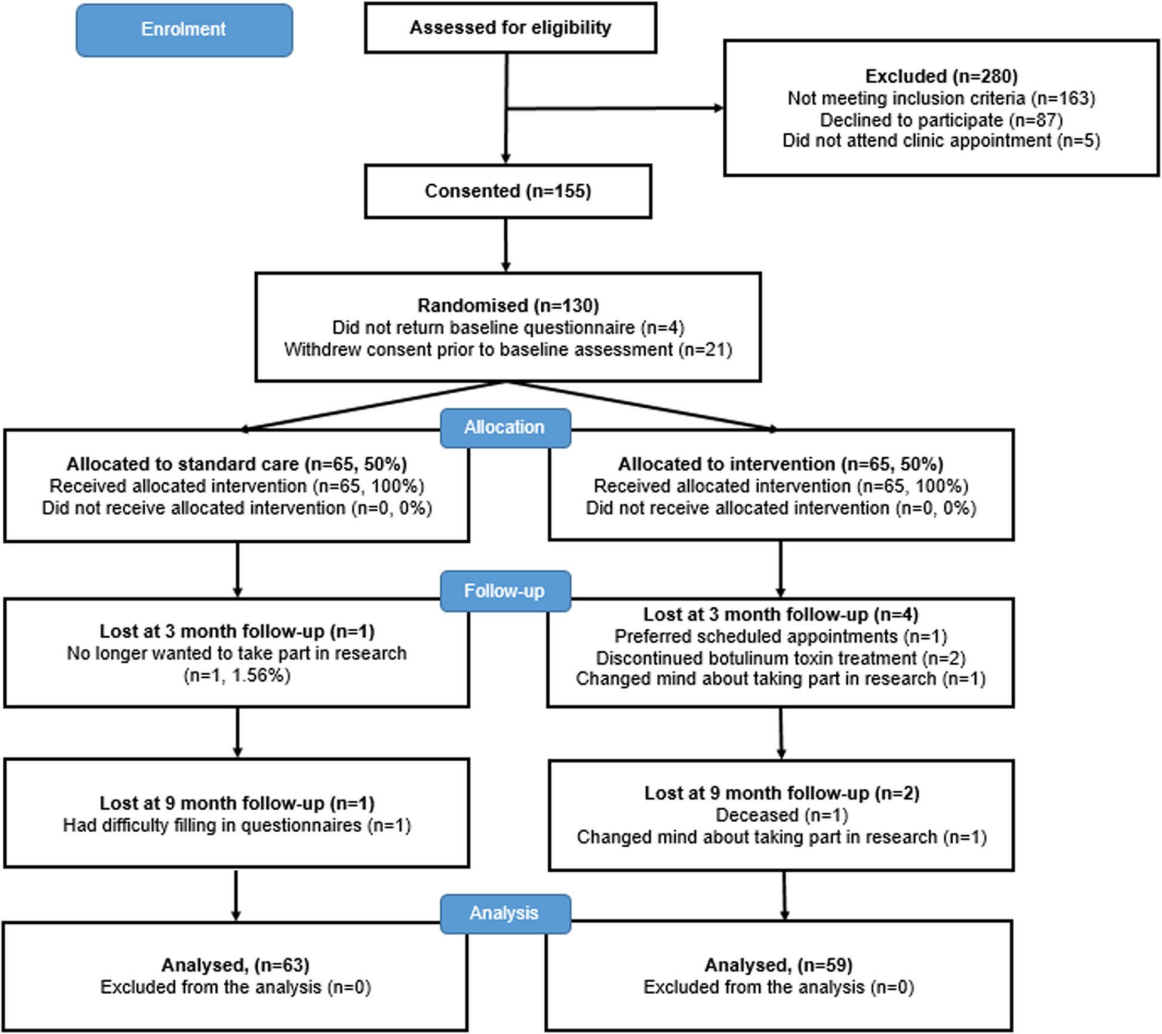
Study flow chart

Of the 410 patients assessed for eligibility, 39.8% (*n* = 163) did not meet the eligibility criteria. Of the 247 eligible patients 5 (2.0%) did not attend their clinic appointment to be approached about the study and 87 (35.2%) declined to participate (Fig. 1). Reasons for declining can be found in Table 1.

**Table 1:** Reasons patients declined to participate

	n (%)
Patient-initiated treatment model deemed more burdensome	8 (9.19)
Not interested in taking part in research or completing questionnaires	29 (33.33)
Not practical to book own appointments	7 (8.05)
Satisfied with current scheduled appointments	41 (47.13)
Thinking about stopping treatment in near future	1 (1.15)
No reason given	1 (1.15)

A total of 155 (62.8%) participants consented to enter the trial. Of these 130 (83.9%) were randomized. Four (2.6%) did not return their baseline assessment and 21 (13.6%) withdrew their consent prior to baseline assessment.

All 130 participants were randomized. A total of 122 participants completed the trial and attended a 9-month follow-up appointment, with 104 (80%) completing the 3 month and 109 (84%) completing the 9-month self-reported questionnaires. Data were analysed for all 122 participants.

### Sample characteristics

Participants’ demographic data are presented in Table 2. A majority of participants were white British and female, with a mean age of 64.0 years (SD = 10.8). There were more patients with BEB (*n* = 77, 59.2%) than HFS (*n* = 53, 40.8%) and the mean duration of BoNT/A treatment was 6.8 years (SD = 6.0). Of the 128 participants with data, 38 (29.7%) reported having a comorbidity. At baseline there were no significant between-group differences in any of the demographic, clinical or psychological measures (Table 2).

**Table 2:** Baseline characteristics

	Intervention (***n*** = 65)	Control (***n*** = 65)
**Age (years), m ± SD**	62.8 ± 10.3	65.1 ± 11.2
**Gender; female, n (%)**	51 (78.5)	42 (64.6)
**Ethnicity, n (%)**		
**Indian**	6 (9.2)	10 (15.4)
**Pakistani**	–	2 (3.1)
**Other Asian background**	4 (6.2)	–
**White British**	43 (66.2)	40 (61.5)
**White Irish**	1 (1.5)	3 (4.6)
**Other White background**	3 (4.6)	–
**Black Caribbean**	2 (3.1)	3 (4.6)
**Black African**	3 (4.6)	1 (1.5)
**Other Black background**	2 (3.1)	–
**Chinese**	1 (1.5)	3 (4.6)
**Other ethnic group**	–	3 (4.6)
**Years in education, m** ± **SD**	13.1 ± 3.3	12.7 ± 3.1
**Employment status, n (%)**		
**Paid employment**	22 (33.8)	21 (32.3)
**Voluntary work**	1 (1.5)	1 (1.5)
**Unemployed**	1 (1.5)	4 (6.2)
**Student**	1 (1.5)	–
**Full time homemaker**	1 (1.5)	4 (6.2)
**Retired**	32 (49.2)	30 (46.2)
**Exempt through disability**	3 (4.6)	2 (3.1)
**Other**	3 (4.6)	2 (3.1)
**Qualifications, n (%)**		
**GCSE /O Level/equivalent**	13 (20)	18 (27.7)
**A Level/equivalent**	6 (9.2)	4 (6.2)
**HNC/HND/equivalent**	5 (7.7)	2 (3.1)
**Degree/equivalent**	19 (29.2)	14 (21.5)
**Postgraduate**	8 (12.3)	7 (10.8)
**Diagnosis, n (%)**		
**BEB**	43 (66.2)	34 (52.3)
**HFS**	22 (33.8)	31 (47.7)
**Disease duration (years), m ± SD**	11.0 ± 6.7	13.8 ± 9.8
**Duration of botulinum toxin type A (months), m ± SD**	72.4 ± 67.0	90.8 ± 76.5
**Number of previous cycles, m ± SD**	19.5 ± 19.3	23.8 ± 23.1
**Last dose of botulinum toxin type A (units), m ± SD**	64.8 ± 60.6	69.8 ± 66.2
**Usual time between treatments (months), m ± SD**	3.2 ± 1.4	3.1 ± 0.9
**Side effects from previous treatment, n (%)**		
**Ptosis**	15 (23.1)	12 (18.5)
**Diplopia**	7 (10.8)	7 (10.8)
**Tearing**	5 (7.7)	8 (12.3)
**Hematoma**	4 (6.2)	5 (7.7)
**Foreign body sensation**	3 (4.6)	7 (10.8)
**Blurred vision**	4 (6.2)	8 (10.8)
**Presence of comorbidities, n (%)**	21 (32.3)	17 (26.2)
**Disease activity, m ± SD**	2.94 ± 2.6	2.74 ± 2.4
**Functional disability, m ± SD**	1.1 ± 1.0	1.1 ± 0.1
**Quality of life - Overall, m ± SD**	28.0 ± 20.4	28.5 ± 21.3
**Quality of life - Activities of daily living, m ± SD**	36.3 ± 24.3	36.7 ± 28.0
**Quality of life - Emotional well-being, m ± SD**	25.3 ± 22.2	23.7 ± 22.1
**Quality of life - Pain, m ± SD**	8.8 ± 11.8	8.43 ± 11.6
**Quality of life - Social/family life, m ± SD**	9.7 ± 12.8	11.90 ± 15.6
**Quality of life - Stigma, m ± SD**	37.0 ± 30.5	37.7 ± 26.7
**Satisfaction with care, m ± SD**	23.8 ± 10.9	23.8 ± 10.7
**Anxiety, m ± SD**	6.8 ± 4.3	6.4 ± 4.8
**Depression, m ± SD**	5.3 ± 4.1	5.19 ± 4.4
**Confidence in the service, m ± SD**	7.3 ± 2.5	6.8 ± 3.2

*M* mean, *SD* standard deviation, *GCSE* General Certificate of Secondary Education, *HNC* Higher National Certificate, *HND* Higher National Diploma, *BEB* Blepharospasm, *HFS* Hemifacial Spasm

### Effects of the intervention

#### Clinical and psychosocial outcomes

The outcome estimates for each variable, by time and group are presented in the table presented in Additional file 1. MLM showed no statistically significant interaction effects on any of the outcomes, except on the HADS anxiety subscale where a significant interaction was found (F _2, 142.4_ = 1.7, *p* = 0.02; Table 3).

**Table 3:** Parameter estimates at each time point for each group and their interaction effect

	Time	Group	Group x Time
**Disease activity**	F _1, 114.7_ = 0.6, *p* = 0.46	F _1, 124.2_ = 0.1, *p* = 0.73	F 1_, 114.7_ = 0.1, *p* = 0.78
**Functional disability**	F _2, 183.0_ = 0.4, *p* = 0.71	F _1, 126.4_ = 0.4, *p* = 0.55	F _2, 183.0_ = 0.5, *p* = 0.61
**Quality of life: Overall score**	F _2, 168.6_ = 0.5, *p* = 0.58	F _1, 127.4_ = 0.2, *p* = 0.62	F _2, 168.6_ = 1.0, *p* = 0.37
**Quality of life: Activities of daily living**	F _2, 188.6_ = 1.8, *p* = 0.16	F _1, 125.4_ = 0.1, *p* = 0.74	F _2, 188.6_ = 0.1, *p* = 0.91
**Quality of life: Emotional wellbeing**	F _2, 151.3_ = 0.1, *p* = 0.95	F _1, 128.9_ < 0.1, *p* = 0.88	F _2, 151.3_ = 1.1, *p* = 0.33
**Quality of life:: Pain**	F _2, 195.7_ = 0.9, *p* = 0.41	F _1, 128.3_ = 0.2, *p* = 0.68	F _2, 195.7_ = 0.9, *p* = 0.41
**Quality of life: Social/family life**	F _2, 171.5_ = 0.9, *p* = 0.41	F _1, 128.4_ = 0.3, *p* = 0.60	F _2, 171.5_ = 1.1, *p* = 0.34
**Quality of life: Stigma**	F _2, 166.9_ = 1.3, *p* = 0.29	F _1, 128.3_ = 0.3, *p* = 0.58	F _2, 166.9_ = 1.0, *p* = 0.37
**Satisfaction with care**	**F**_**2, 203.0**_ **= 5.9,** ***p*** **= 0.03**	F _1, 113.1_ = 0.5, *p* = 0.83	F _2, 203.0_ = 1.4, *p* = 0.25
**Anxiety**	F _2, 142.3_ = 1.6, *p* = 0.20	F _1, 130.0_ = 0.1, *p* = 0.75	**F**_**2, 142.3**_ **= 1.6,** ***p*** **= 0.02**
**Depression**	F _2, 165.8_ = 1.4, *p* = 0.26	F _1, 130.6_ = 0.5, *p* = 0.50	F _2, 165.8_ = 1.2, *p* = 0.31
**Confidence in the service**	F _2, 205.1_ = 1.6, *p* = 0.20	F _1, 116.3_ = 2.9, *p* = 0.09	F _2, 205.1_ = 0.02, *p* = 0.98

**Fig. 2:**
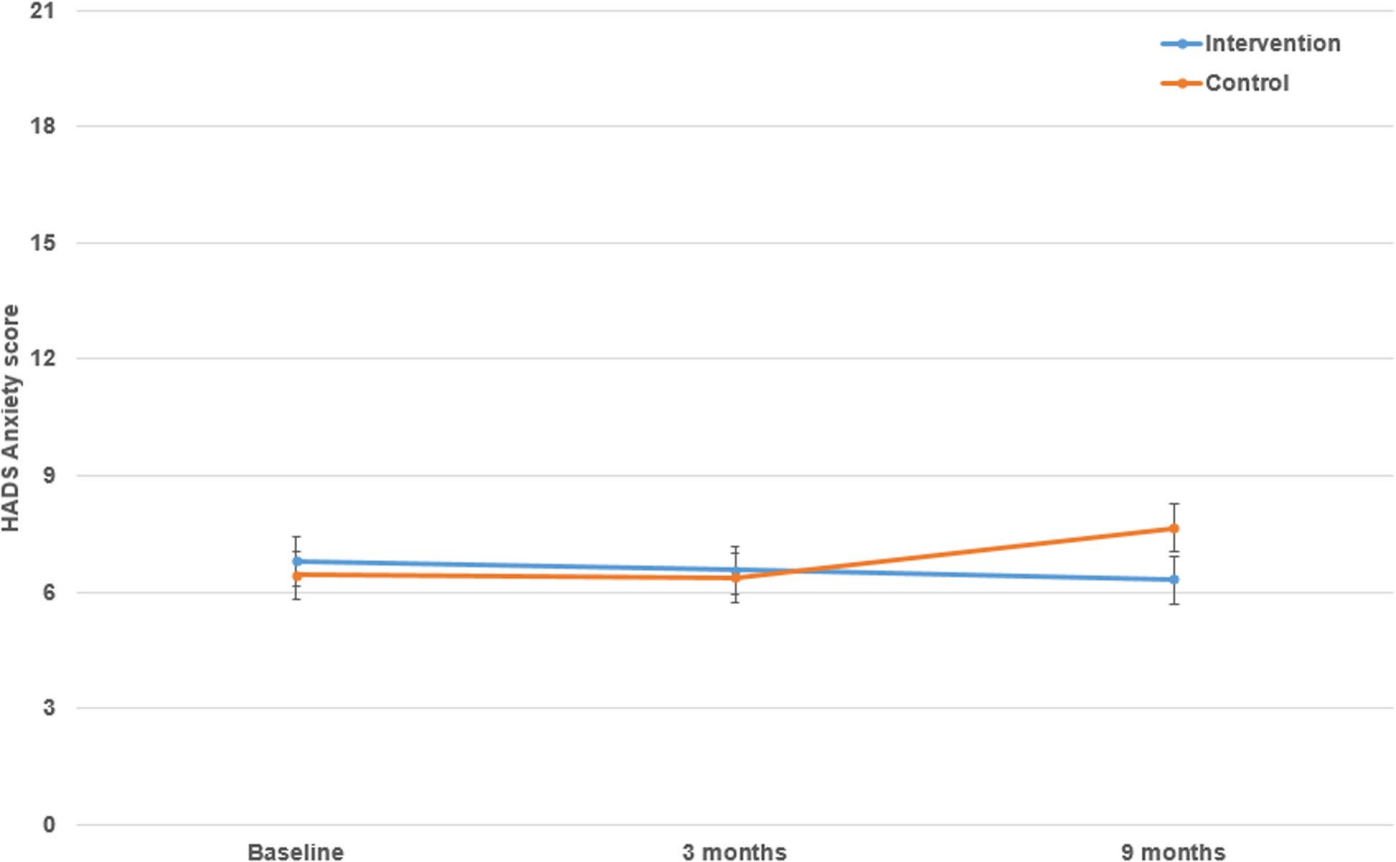
EMM for HADS Anxiety scores overtime

Estimated marginal means (EMM) suggest that levels of anxiety increased in the control arm and declined in the intervention arm (Fig. 2). An additional file shows this in more detail (Additional file 1); where the largest change in anxiety scores between the two groups was observed between the 3- and 9- month follow-ups. Inspection of the EMM indicated that anxiety scores remained within the normal range (score 0-7) for each group at all time points. The effect size at final follow-up was small (Hedges’ g = − 0.26 [99% CI -0.83, 0.32]).

#### Healthcare usage

On average participants in both groups made two visits to the nurse-led clinic during the trial period (Control Grp SD = 1.0; Intervention Grp SD = 1.4). Poisson regression showed that group was not a predictor of number of treatments received during the trial even after adjusting for age, sex, type of diagnosis, year of diagnosis, and years of education (Exp(β) = 1.022, χ^2^ (0.03, *n* = 113); df = 1, *p* = 0.86). There was also no significant difference between groups on the number of days between clinic visits (Control Grp mean = 95.36, SD = 33.14; Intervention Grp mean = 100.5, SD = 54.5; F (116) = 1.3, *p* = 0.53).

#### Costs

Table 4 shows the total cost per case, total costs for the health service, primary and secondary care and costs to individuals between study arms. Mean total costs of care per intervention participant over the trial period was £832.22 (SD = £993.36) and for control participants £1031.18 (SD = £1302.54). This suggests that usual care was £198 more expensive per participant (95% CI -£654.67, £256.76), however this difference was not statistically significant (t (96) = 0.86, *p* = 0.39).

**Table 4:** Cost per patient by treatment arm (£ Pound sterling)

	Control			Intervention			Mean difference	95% CI of the difference		***p***
	M	SD	% of cost per case	M	SD	% of cost per case				
**Primary care**	19.15	35.56	1.86	13.37	24.43	1.61	−5.78	−16.62	5.06	0.30
*General Practitioner*	11.73	29.06	1.14	10.87	21.99	1.31	−0.86	−9.94	8.22	0.85
*Other community services*	3.63	8.88	0.35	2.50	7.47	0.30	−1.13	−3.93	1.85	0.48
**Secondary care**	801.73	111.16	77.75	608.10	111.16	73.07	−193.63	−521.78	134.53	0.25
*Nurse-led clinic*^a^	260.69	150.66	25.28	216.10	156.98	25.97	−44.59	− 102.60	13.42	0.13
*Other outpatient services*	75.78	111.16	7.35	73.19	108.48	8.79	−2.60	−44.76	39.57	0.90
*Inpatient stays*	488.42	1184.02	47.36	308.21	839.69	37.03	− 180.21	− 538.17	177.76	0.32
*Accident & emergency*	4.23	34.11	0.41	12.72	46.98	1.53	8.49	−5.62	22.61	0.24
**Total cost to the health service**	840.20	111.16	81.48	629.37	111.16	75.63	− 210.83	− 542.29	120.63	0.21
**Individual costs**	321.33	414.39	31.16	294.31	364.93	35.36	−27.03	−178.19	124.14	0.73
*Personal costs*	288.78	421.41	28.01	246.50	364.86	29.62	−42.28	− 194.30	109.73	0.59
*Travel costs*	43.55	53.27	4.22	53.26	53.46	6.40	9.72	−9.98	29.41	0.33
**Total cost per case**	1031.18	1302.54		832.22	993.36		−198.95	−654.67	256.76	0.39

*M* Mean, *SD* standard deviation, *CI* confidence interval

^a^including treatment and visit

The average cost of primary care was higher in the control compared to intervention arm by £13.37 (95% CI -£16.62, £5.06). Secondary care costs represented approximately 75% of the overall costs in both arms and was lower in the intervention compared to control group by £193.63 (95% CI -£521.78, £134.53). This was largely due to reduced inpatient stays in the intervention arm. The costs of delivering primary (t (103.60) = 1.05, *p* = 0.30) and secondary care (t (125) = 1.16, *p* = 0.25) services however did not differ significantly between arms.

The cost to individual participants, in terms of personal and travel costs, was £27.03 (95% CI -£178.19, £124.14) greater, on average, in the control versus intervention arm. This difference was not statistically significant (*p* = 0.73). There were also no significant differences between groups on the average number of employment days lost due to dystonia, in those that were working either voluntarily or in paid employment (Intervention Grp mean = 1.60 SD = 2.34; Control Grp mean = 2.58, SD = 6.27, t (35) = 0.36, *p* = 0.53).

## Discussion

The findings of this RCT indicate that a patient-initiated treatment model for people with BEB or HFS receiving BoNT/A was not superior to usual care on any the primary outcomes. However, this new model did not have a negative impact on disease severity, functional disability, or depression. This is consistent with the results of previous RCTs evaluating similar services in other long-term conditions [4, 5, 23, 24] and supports the notion that there is little to no risk of harm when patients have greater control of their treatment regimen. Patients receiving this model of care were equally satisfied with the service and confident in their care as those receiving treatment as usual.

Notably, anxiety reduced in the patient-initiated treatment model as opposed to increasing for participants in the control arm, albeit scores remained within the normal range. The small increase in anxiety scores in the usual care group may reflect the common discomfort and fluctuations in mood experienced by people with dystonia when symptoms return after treatment [7, 25], particularly if symptoms return well in advance of a fixed follow-up appointment. This pattern also replicates that of other patient-initiated services [23–27], suggesting that by empowering patients to control the timing of their own treatment, this can reduce the anxiety associated with the burden of cyclical treatments. This also challenges the view that anxiety may increase if patients are more involved and responsible for initiating their own treatment. As this model of care did not directly target depression or anxiety it would have been unrealistic to expect significant improvements in mood over the trial period.

Responding to the need for economic evaluations of patient-initiated services [5] this trial explored the costs of delivering the service and financial costs to the patient. Whilst the intervention was not cost saving at a statistically significant level, it could be argued that a patient-initiated treatment model might save NHS resources given the slightly reduced costs found in the intervention arm. The wide confidence intervals around the mean differences however suggest poor precision and low statistical power. It is therefore possible that our study was statistically underpowered to detect between-group differences on our primary outcome measures. On closer inspection of the economic data there are aspects of care that have some notable differences in costs, despite a lack of between-group differences. For example, secondary care costs, which accounted for a majority of the overall costs per participant, were on average £193.63 more expensive for those in the control arm. Although data on the specific nature of these inpatient stays were not available, they are unlikely to be associated with an individual’s dystonia. The costs of operating the nurse-led clinic, including the medication, were marginally lower in the intervention compared to control arm but not statistically different. Again, confidence intervals indicate there was significant variance in this estimate and given a larger sample could have been statistically significant.

The data suggest that when patients with BEB or HFS are able to initiate their own treatment schedule they do so at a similar rate and frequency as healthcare professionals booking follow-up appointments on their behalf. This could reflect the usual care service responding appropriately to the needs of the population at this center or a group of patients who are entrenched in a system of periodic appointments. Considering the average disease duration was 12 years and patients had been receiving treatment for on average 7 years, this may well be the case.

A “one size fits all” approach to patients managing their condition and treatment may not be appropriate [28] and consideration must be made for those who preferred the traditional model of care. Participation rates show that most of the patients approached agreed to take part, reflecting figures in other trials of patient-initiated services [23–27, 29] There was however, a 46% refusal rate, which, if this number were to persist when not under trial circumstances, suggests that further work is needed prior to any widespread implementation. Refusal to participate in trials that increase patient involvement in healthcare have been linked to a reluctance to disrupt services and relationships that are working well and are highly valued [30]. In the context of BoNT/A treatment for BEB, a recent study found 64% of patients were satisfied with a treatment schedule of between 8 and 12 weeks [31]. Preference for the fixed 12-week schedule was reflected in the reasons patients gave for not participating in this trial. Qualitative analysis suggests that for patient-initiated treatment models to be successfully implemented, patients need to have confidence in the system and the speed at which appointments can be accessed [32]. Data from the study however suggests that participants were equally as confident in this new model of care as treatment as usual, and could meet the needs of over a third of patients who would prefer a shorter or longer treatment schedule than the typical 12 weeks. A more in-depth understanding of the acceptability of the new treatment model, has been further analyzed in a qualitative study and is reported elsewhere [33]. The views of healthcare professionals within the dystonia service would also be valuable to future implementation efforts and an in-depth understanding of their beliefs could be obtained from future research.

There are some limitations that need to be noted. We did not collect data on the number and duration of telephone calls made to the triage service. Addition of these costs could have a significant impact on healthcare usage and cost data as they may constitute a substantial time burden on staff. The inability to blind participants and healthcare professionals may have also biased the results of the evaluation [34, 35]. The use of a patient-reported scale (BSDI) as a primary outcome measure could be viewed as a limitation of a trial where patients were not blind to study group. However, this was used in combination with other rating scales addressing functional impairment, following the practice of clinical studies in this area [36, 37]. Furthermore, the single-center status of the trial has reduced the external validity of the findings [38] and may have led to larger intervention effects [39]. There is also the possibility that a trial of longer duration (e.g., 12 months or beyond) might have revealed significant differences in clinical, psychological and cost outcomes due to more involvement of the healthcare service. A larger multicenter RCT of longer duration would therefore be required in order to test our hypothesis more robustly.

## Conclusions

In this RCT, we did not observe differences between the patient-initiated treatment model and usual care for patients receiving BoNT/A on measures of BEB and HFS severity or disability. There were also no differences between the two groups in quality of life, depression, or satisfaction and confidence with the service. The findings did suggest that enabling patients to control the timing of their treatment could better control anxiety. Overall, the findings in this study suggest that the patient-initiated appointments and usual care were equally effective and have a similar impact on patient reported outcomes with a group whose treatment was stable. Modest cost savings could potentially be made at the individual level and to the NHS however this requires further exploration on a larger scale. In the context of the general increased attitude and practice to involve patients in decisions regarding their treatment, it would be valuable to establish whether patient-initiated treatment has any specific advantages to patients compared to usual care in patients with dystonia receiving BoNT/A for outcomes that were not explored in this study including acceptability from the perspective of patients and healthcare professionals.

## Supplementary Information

**Additional file 1.** Table of estimated marginal means for each variable at each time point.

## Data Availability

Anonymized data from the current study can be made available, if approved by our REC.

## Abbreviations

BEB: Blepharospasm
BSDI: Blepharospasm Disability Index
CDQ-24: Craniocervical Dystonia Questionnaire
CSRI: Client Service Receipt Inventory
CSQ: Client Satisfaction Questionnaire
HADS: Hospital Anxiety & Depression Scale
HFS: Hemifacial spasm
JRS: Jankovic Rating Scale
NHS: National Health Service
RCT: Randomised controlled trial
SD: Standard deviation
UK: United Kingdom
