# Supplementary material for: A patient-initiated treatment model for blepharospasm and hemifacial spasm: a randomized controlled trial
Source: BMC Neurol. 2022 Mar 17;22:99. doi: 10.1186/s12883-022-02603-7 (PMC8928587; doi:10.1186/s12883-022-02603-7)
Supplement: Supplementary file 1 — Additional file 1. Table of estimated marginal means for each variable at each time point. [file 12883_2022_2603_MOESM1_ESM.pdf]

Table of estimated marginal means for each variable at each time point

| | Baseline | | | | | 3 months | | | | | 9 months | | | | |
|---|---|---|---|---|---|---|---|---|---|---|---|---|---|---|---|
| | **M** | **SE** | **df** | **95% CI** | | **M** | **SE** | **df** | **95% CI** | | **M** | **SE** | **df** | **95% CI** | |
| **Disease activity** | | | | | | | | | | | | | | | |
| **Intervention** | 2.94 | 0.30 | 211.67 | 2.34 | 3.53 | - | - | - | - | - | 3.06 | 0.34 | 225.37 | 2.39 | 3.73 |
| **Control** | 2.74 | 0.30 | 211.67 | 2.14 | 3.33 | - | - | - | - | - | 3.01 | 0.33 | 221.52 | 2.37 | 3.66 |
| | **Effect size (99% CI)** | | | | | **-** | | | | | **0.02 (-0.49, 0.52)** | | | | |
| **Functional disability** | | | | | | | | | | | | | | | |
| **Intervention** | 1.05 | 0.12 | 172.63 | 0.80 | 1.29 | 0.99 | 0.13 | 212.31 | 0.73 | 1.25 | 1.02 | 0.13 | 204.34 | 0.76 | 1.27 |
| **Control** | 1.07 | 0.12 | 172.63 | 0.83 | 1.31 | 1.08 | 0.13 | 194.81 | 0.83 | 1.34 | 1.18 | 0.13 | 192.45 | 0.93 | 1.43 |
| | **Effect size (99% CI)** | | | | | **-0.10 (-0.62, 0.43)** | | | | | **-0.16 (-0.70, 0.39)** | | | | |
| **Quality of life: Overall** | | | | | | | | | | | | | | | |
| **Intervention** | 28.03 | 2.60 | 170.22 | 22.90 | 33.16 | 27.23 | 2.78 | 207.68 | 21.74 | 32.71 | 24.68 | 2.76 | 201.13 | 19.23 | 30.12 |
| **Control** | 28.50 | 2.60 | 170.22 | 23.38 | 33.63 | 27.66 | 2.70 | 190.93 | 22.34 | 32.98 | 28.80 | 2.70 | 189.50 | 23.48 | 34.12 |
| | **Effect size (99% CI)** | | | | | **-0.02 (-0.53, 0.49)** | | | | | **-0.19 (-0.74, 0.37)** | | | | |
| **Quality of life – activities of daily living** | | | | | | | | | | | | | | | |
| **Intervention** | 36.25 | 3.14 | 176.16 | 30.05 | 42.45 | 33.88 | 3.41 | 218.51 | 27.16 | 40.60 | 32.04 | 3.34 | 208.30 | 25.44 | 38.63 |
| **Control** | 36.76 | 3.14 | 176.16 | 30.56 | 42.95 | 35.43 | 3.29 | 200.13 | 28.95 | 41.91 | 34.10 | 3.26 | 196.43 | 27.67 | 40.54 |
| | **Effect size (99% CI)** | | | | | **-0.06 (-0.57, 0.46)** | | | | | **-0.08 (-0.60, 0.44)** | | | | |
| **Quality of life: Emotional well-being** | | | | | | | | | | | | | | | |
| **Intervention** | 25.32 | 2.79 | 175.04 | 19.82 | 30.82 | 24.81 | 2.98 | 212.67 | 18.94 | 30.68 | 23.34 | 2.99 | 207.79 | 17.45 | 29.23 |
| **Control** | 23.65 | 2.79 | 175.04 | 18.15 | 29.16 | 24.74 | 2.89 | 195.73 | 19.04 | 30.44 | 26.75 | 2.91 | 195.65 | 21.02 | 32.48 |
| | **Effect size (99% CI)** | | | | | **<0.01 (-0.50, 0.50)** | | | | | **-0.14 (-0.68, 0.40)** | | | | |
| **Quality of life: Pain** | | | | | | | | | | | | | | | |
| **Intervention** | 8.17 | 1.41 | 209.64 | 5.38 | 10.96 | 9.25 | 1.58 | 253.35 | 6.14 | 12.36 | 6.51 | 1.54 | 248.39 | 3.48 | 9.54 |
| **Control** | 8.43 | 1.42 | 209.64 | 5.64 | 11.22 | 8.90 | 1.50 | 236.16 | 5.93 | 11.86 | 8.78 | 1.49 | 235.39 | 5.84 | 11.72 |
| | **Effect size (99% CI)** | | | | | **0.03 (-0.48, 0.54)** | | | | | **-0.18 (-0.74, 0.37)** | | | | |
| **Quality of life: Social Family Life** | | | | | | | | | | | | | | | |

| | Baseline | | | | | 3 months | | | | | 9 months | | | | |
|---|---|---|---|---|---|---|---|---|---|---|---|---|---|---|---|
| | M | SE | df | 95% CI | | M | SE | df | 95% CI | | M | SE | df | 95% CI | |
| **Intervention** | 9.66 | 1.76 | 180.90 | 6.19 | 13.13 | 10.39 | 1.90 | 222.57 | 6.64 | 14.14 | 10.59 | 1.89 | 214.87 | 6.87 | 14.31 |
| **Control** | 11.90 | 1.76 | 180.90 | 8.43 | 15.37 | 9.80 | 1.84 | 204.33 | 6.18 | 13.42 | 12.49 | 1.84 | 202.47 | 8.87 | 16.11 |
| | **Effect size (99% CI)** | | | | | **0.04 (-0.47, 0.55)** | | | | | **-0.13 (-0.66, 0.41)** | | | | |
| **Quality of life: Stigma** | | | | | | | | | | | | | | | |
| **Intervention** | 37.00 | 3.62 | 179.33 | 29.85 | 44.15 | 35.15 | 3.91 | 220.03 | 27.44 | 42.86 | 30.66 | 3.88 | 213.19 | 23.00 | 38.31 |
| **Control** | 37.65 | 3.62 | 179.33 | 30.50 | 44.80 | 36.10 | 3.78 | 202.12 | 28.65 | 43.55 | 36.94 | 3.78 | 200.78 | 29.49 | 44.40 |
| | **Effect size (99% CI)** | | | | | **-0.03 (-0.54, 0.48)** | | | | | **-0.20 (-0.76, 0.35)** | | | | |
| **Satisfaction with care** | | | | | | | | | | | | | | | |
| **Intervention** | 23.83 | 0.99 | 302.27 | 21.89 | 25.78 | 26.37 | 1.19 | 320.59 | 24.03 | 28.71 | 21.13 | 1.12 | 307.32 | 18.92 | 23.34 |
| **Control** | 23.75 | 0.99 | 302.27 | 21.80 | 25.69 | 25.02 | 1.14 | 319.51 | 22.77 | 27.26 | 23.21 | 1.07 | 305.84 | 21.10 | 25.32 |
| | **Effect size (99% CI)** | | | | | **0.14 (-0.40, 0.68)** | | | | | **-0.23 (-0.80, 0.33)** | | | | |
| **Anxiety** | | | | | | | | | | | | | | | |
| **Intervention** | 6.79 | 0.61 | 160.34 | 5.59 | 7.99 | 6.56 | 0.64 | 187.84 | 5.30 | 7.82 | 6.32 | 0.64 | 186.81 | 5.06 | 7.59 |
| **Control** | 6.44 | 0.61 | 160.34 | 5.24 | 7.64 | 6.37 | 0.62 | 175.11 | 5.14 | 7.60 | 7.65 | 0.63 | 176.46 | 6.41 | 8.89 |
| | **Effect size (99% CI)** | | | | | **0.04 (-0.47, 0.55)** | | | | | **-0.26 (-0.83, 0.32)** | | | | |
| **Depression** | | | | | | | | | | | | | | | |
| **Intervention** | 5.26 | 0.53 | 178.43 | 4.21 | 6.31 | 4.87 | 0.57 | 218.25 | 3.74 | 6.00 | 5.22 | 0.57 | 210.40 | 4.10 | 6.34 |
| **Control** | 5.19 | 0.53 | 178.43 | 4.14 | 6.24 | 5.48 | 0.55 | 200.62 | 4.39 | 6.58 | 6.09 | 0.55 | 198.67 | 5.00 | 7.18 |
| | **Effect size (99% CI)** | | | | | **-0.13 (-0.67, 0.40)** | | | | | **-0.19 (-0.74, 0.36)** | | | | |
| **Confidence in the service** | | | | | | | | | | | | | | | |
| **Intervention** | 7.34 | 0.33 | 319.08 | 6.69 | 7.99 | 7.82 | 0.39 | 326.61 | 7.06 | 8.58 | 7.34 | 0.38 | 323.62 | 6.60 | 8.08 |
| **Control** | 6.76 | 0.33 | 319.08 | 6.11 | 7.41 | 7.35 | 0.36 | 324.59 | 6.64 | 8.06 | 6.74 | 0.36 | 322.77 | 6.03 | 7.44 |
| | **Effect size (99% CI)** | | | | | **0.15 (-0.39, 0.70)** | | | | | **0.20 (-0.35, 0.76)** | | | | |

M: Mean SE: Standard error, df: degrees of freedom, CI: confidence interval
